# Spatial Transcriptomics: Emerging Technologies in Tissue Gene Expression Profiling

**DOI:** 10.1021/acs.analchem.3c02029

**Published:** 2023-10-10

**Authors:** Agustín Robles-Remacho, Rosario M. Sanchez-Martin, Juan J. Diaz-Mochon

**Affiliations:** † GENYO. Centre for Genomics and Oncological Research, Pfizer/University of Granada/Andalusian Regional Government, PTS Granada, Avenida de la Ilustracion, 114. 18016 Granada, Spain; ‡ Department of Medicinal and Organic Chemistry, School of Pharmacy, University of Granada, Campus Cartuja s/n, 18071 Granada, Spain; § Instituto de Investigación Biosanitaria ibs.GRANADA, 18012 Granada, Spain

## Abstract

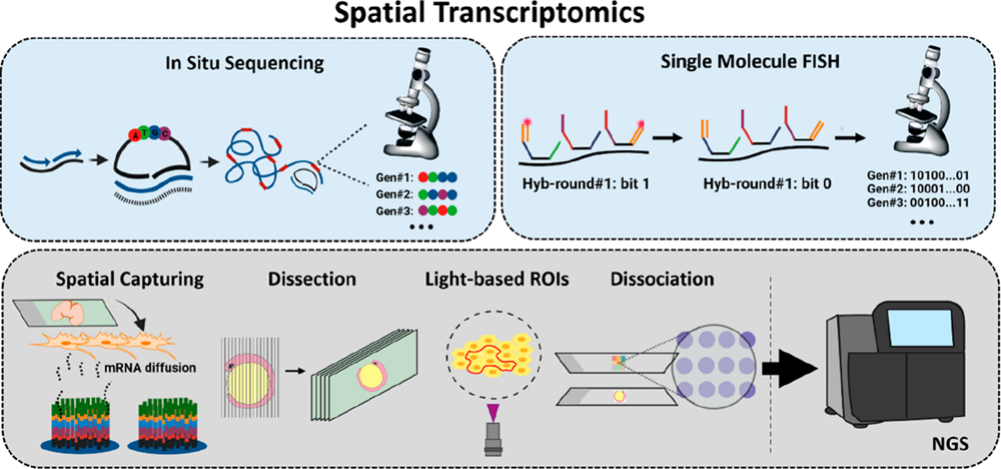

In this Perspective, we discuss the current status and advances in spatial transcriptomics technologies, which allow high-resolution mapping of gene expression in intact cell and tissue samples. Spatial transcriptomics enables the creation of high-resolution maps of gene expression patterns within their native spatial context, adding an extra layer of information to the bulk sequencing data. Spatial transcriptomics has expanded significantly in recent years and is making a notable impact on a range of fields, including tissue architecture, developmental biology, cancer, and neurodegenerative and infectious diseases. The latest advancements in spatial transcriptomics have resulted in the development of highly multiplexed methods, transcriptomic-wide analysis, and single-cell resolution utilizing diverse technological approaches. In this Perspective, we provide a detailed analysis of the molecular foundations behind the main spatial transcriptomics technologies, including methods based on microdissection, *in situ* sequencing, single-molecule FISH, spatial capturing, selection of regions of interest, and single-cell or nuclei dissociation. We contextualize the detection and capturing efficiency, strengths, limitations, tissue compatibility, and applications of these techniques as well as provide information on data analysis. In addition, this Perspective discusses future directions and potential applications of spatial transcriptomics, highlighting the importance of the continued development to promote widespread adoption of these techniques within the research community.

## 1 Introduction

### 1.1 The Importance of the Spatial Detection of RNA

Spatial transcriptomics began gaining traction after Stahl et al.’s seminal 2016 paper.^1^ These technologies, which retain RNA’s spatial distribution within tissues, have advanced our understanding of transcriptomes’ spatial organization in biological mechanisms.^2−6^ Although effective, RNA-seq methods require extraction and homogenization of the RNA content, losing spatial gene expression information, essential for some investigations and pathologies.^2−6^ Single-cell RNA-sequencing (scRNA-seq) offers high-resolution individual cell analysis but lacks spatial resolution.^7^ Combining spatially resolved and bulk RNA-sequencing methods can provide valuable insights into complex biological systems.^8−11^

Spatially resolved RNA technologies hold promise in cancer research, biomarker discovery, and drug development,^12^ especially as more RNA molecules become potential small molecule drug targets.^13^ They help address challenges posed by intratumoral heterogeneity by identifying and mapping subclone territories in tumors.^14,15^ Spatial techniques have been widely used in neurosciences to study specific brain cell types^16^ and have mapped gene expression patterns in Alzheimer’s disease.^17^ In developmental biology, these techniques help understand organ and tissue formation and map transcriptomes in Human Cell Atlases.^15,18^ Spatial techniques also identify infected cell types in infectious diseases.^19^ Overall, spatial transcriptomics significantly impacts generating accurate molecular profiles based on RNA signatures.

### 1.2 Techniques for RNA Detection with Spatial Resolution: Nonbarcoded smFISH, LCM, and Tomo-seq

**Figure 1:**
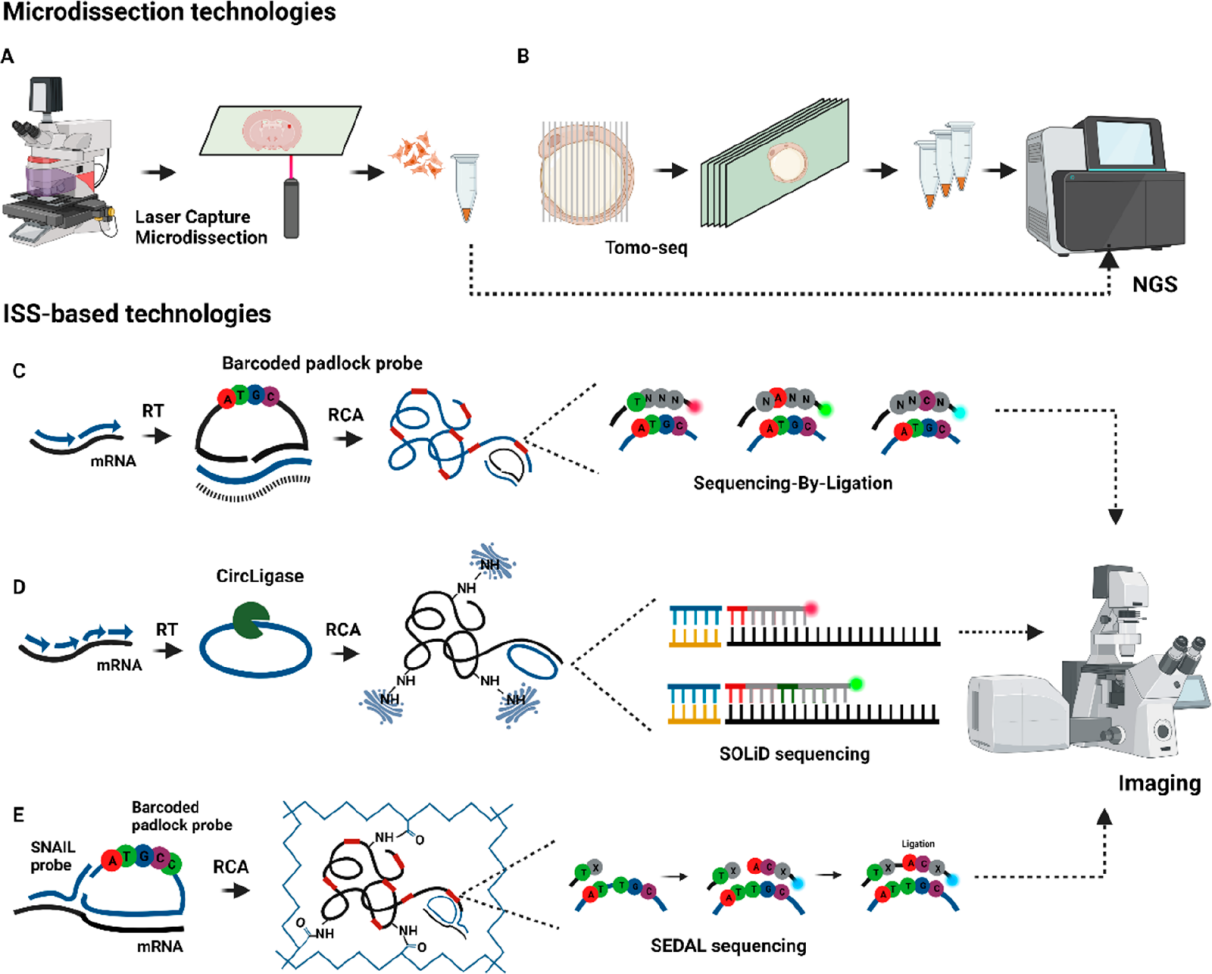
Microdissection and ISS-based technologies. (A) LCM is used to dissect single cells or small areas, while in (B) Tomo-seq a zebrafish embryo is dissected in multiple axes. After dissection, the samples are processed for library preparation and NGS. (C) In ISS, mRNA is retrotranscribed to cDNA followed by the hybridization of barcoded padlock probes. After ligation, the probes are amplified via RCA and decoded using SBL. (D) FISSEQ circularizes cDNA using CircLigase, followed by RCA and sequencing using SOLiD. (E) In STARmap, a SNAIL probe and a padlock probe allow for RCA without an RT step, followed by SEDAL sequencing.

Singer et al. conducted the first *in situ* gene expression study in 1982 and developed the single-molecule RNA fluorescence *in situ* hybridization (smRNA-FISH) method in 1998.^20,21^ This method was later extended as smFISH,^22^ a nonbarcoded method that employs a collection of 40–50 probes designed to target a single mRNA species, enabling the simultaneous detection of a reduced number of mRNA species at their respective subcellular locations, limited by the number of distinct fluorophores used. Technologies like RNAscope,^23^ another nonbarcoded smFISH method, also allow for the detection of a limited number of mRNA species and have demonstrated their usefulness and efficiency in different biological studies.^24^ Microdissection-based technologies, such as laser-capturing microdissection (LCM), have been widely used due to easy implementation. LCM, developed in the late 20th century, isolates specific cells or regions of interest (ROIs) for subsequent PCR amplification and RNA-sequencing (Figure 1A).^25^ However, LCM is laborious and limited in the number of cells analyzed from a tissue. Tomo-seq, another dissection technology that has spread in research, involves creating micrometric sections for RNA-sequencing (Figure 1B) for posterior analysis and RNA-sequencing. Tomo-seq was first developed on 18 μm sections of zebrafish embryos,^26^ and since then it has been adopted in various biological systems.^27,28^ Nevertheless, its main limitation is that the resolution of transcriptomic data is restricted by the section thickness, achieving up to 8 μm,^28^ lacking single-cell resolution.

Since then, new spatial RNA methods with high multiplexing capacity, transcriptomic-wide analysis, and single-cell resolution have emerged. In this work, we provide perspective on methods that enable the simultaneous detection of at least 100 different mRNA species, and we have grouped them into six categories based on (1) microdissection, (2) *in situ* sequencing, (3) barcoded smFISH, (4) spatial capturing, (5) ROI selection, and (6) single cell or nuclei dissociation as barcoded spots. We review the molecular principles behind these methods and provide their detection efficiency, generally considering nonbarcoded smFISH sensitivity as 100% or providing the number of unique molecular identifiers (UMIs) or genes detected per area. In addition, we review the strengths, limitations, and tissue compatibility. Additionally, we discuss future directions and potential applications of spatial transcriptomics.

## 2 *In Situ* Sequencing-Based Technologies

### 2.1 ISS

In 2013, Nilsson’s lab published *in situ* sequencing (ISS) for sequencing gene expression in fresh frozen tissues.^29^ ISS involves tissue fixation, reverse transcription (RT) of mRNA into complementary DNA (cDNA), and targeting cDNA using padlock probes. Following hybridization, the probes undergo circularization, ligation, and amplification using rolling circle amplification (RCA), resulting in the generation of DNA nanoballs. To identify each mRNA species, a barcoding strategy is carried out. The barcoding strategy is enabled by sequencing either unique four-nucleotide barcodes on the padlock probe or leaving a four-nucleotide gap in target cDNA. In both cases, the barcode is amplified through RCA and decoded using sequencing-by-ligation (SBL) (Figure 1C). SBL employs an anchor primer to bind near the target sequence and ligates a fluorescently labeled detection probe. These probes are divided into four libraries with four distinct fluorophores, each probe with one fixed base and eight random positions. Imaging is conducted to identify the best-matching probe and determine the fixed base. This process is repeated four times, resulting in a four-nucleotide barcode linked to a specific mRNA species.

ISS has been used to detect up to 222 gene transcripts^17^ in a variety of tissues for architecture, development, and disease research.^15,30^ The method’s main advantage is its robustness, with a detection efficiency of 5%–30%,^2,31^ and it has been commercialized by 10X Genomics. The main limitations are the ability to detect only predetermined targets and a moderate capacity for multiplexing, partly attributed to the size of the amplicons within the cell, causing optical crowding. In 2020, the group developed hybridization-based ISS (HybISS), improving the signal-to-background ratio (SBR) 5-fold using sequencing-by-hybridization (SBH) in human and mouse brain tissues.^32^ In 2020, SCRINSHOT was introduced as a method to avoid the RT step that typically reduces efficiency, employing SplintR ligase to directly ligate padlock probes to the RNA content, showing improved efficiency compared to cDNA-based ISS.^33^ In 2021, HybRISS was published, using chimeric padlock probes to directly ligate RNA molecules in mouse coronal brain tissue sections, avoiding the RT step and improving efficiency 5-fold compared to ISS.^34^

### 2.2 FISSEQ

In 2014, George Church’s group published fluorescent *in situ* sequencing (FISSEQ).^35,36^ In FISSEQ, mRNA is retro-transcribed to cDNA in fixed cells, followed by *in situ* circularization of the cDNA using CircLigase. Aminoallyl-dUTPs are used to add primary amines to cDNA fragments during RT. RCA amplifies the cDNA fragments to create DNA nanoballs. The nanoballs are cross-linked using BS(PEG)_9_ to form a porous matrix, and SBL is used as a barcoding strategy to decode up to 30 base pairs. FISSEQ detected ∼8,000 mRNA species in 40 primary fibroblast cells,^36^ enabling *de novo* transcriptomics analyses. However, it has a low efficiency (0.001%)^11,36^ due to rRNA interference during mRNA detection (Figure 1D).

In 2021, ExSeq was developed, combining FISSEQ and expansion microscopy.^37^ It improved the detection efficiency to 60% in a 15 μm mouse hippocampus slice. ExSeq employs expansion microscopy chemistry to separate RNAs, avoiding optical crowding, and incorporates *ex situ* analysis by extracting cDNA amplicons for next generation sequencing (NGS). It can be used for both *de novo* and targeted analyses using padlock probes. Also in 2021, BOLORAMIS was developed, combining FISSEQ, padlock probes, and SplintR ligase for direct ligation of padlock probes onto RNA molecules in brain cell cultures.^38^ By avoiding the RT step, BOLORAMIS accessed more transcript species than mRNA as noncoding transcripts.^38^

### 2.3 STARmap

Spatially resolved transcript amplicon readout mapping (STARmap), published in 2018 by Deisseroth’s group, was employed for studying 3D tissue sections embedded in a hydrogel tissue.^39^ It uses SNAIL probes (Specific amplification of Nucleic Acids via Intramolecular Ligation) that ligate first mRNA. Then the SNAIL probe is targeted by a padlock probe, followed by ligation and RCA, thus avoiding the RT step. As a barcoding strategy, each mRNA is associated with a five-barcode allocated in the padlock probe. This barcode is decoded using a modified version of SBL known as sequencing with error-reduction by dynamic annealing and ligation (SEDAL). SEDAL uses two types of probes: an anchor probe with a fixed position and degenerate bases and decoding probes with fluorescent tags, two fixed nucleotides, and additional degenerate bases. Ligation happens only when both probes perfectly match the DNA template (Figure 1E).

STARmap was used to detect 1020 RNA species in mouse brain sections with efficiency comparable to scRNA-seq.^2,39^ In 3D cubic sections (150 μm thick), it detected 28 genes in over 30,000 cells.^39^ Although limited to 28 genes in thick sections, STARmap’s strength lies in its high multiplexing capacity and ability to resolve thick tissue sections. In 2023, STARmap PLUS detected 2,766 targeted genes and two pathologic biomarker proteins across various mouse brain tissues in an Alzheimer’s mouse model.^40^

In summary, the ISS is robust in target detection with an efficiency ranging from 5% to 30%. However, it has limitations in detecting predetermined targets and has a moderate multiplexing capacity (222 gene transcripts) attributed to the size of the amplicons that cause optical crowding. HybISS adaptation enhanced the SBR ratio compared to ISS. FISSEQ allows for *de novo* transcriptomics analyses but suffers from low efficiency due to rRNA interference. ExSeq advancements enhance FISSEQ detection efficiency and multiplexing capacity, and SCRINSHOT, HybRISS, BOLORAMIS, and STARmap enable direct detection of RNA content, bypassing the RT step that typically reduces efficiency with the possibility to expand to more species than mRNA. STARmap has a valuable high multiplexing capacity and can resolve thick tissue sections. However, it may be limited in the number of transcripts detected in thicker sections (28 gene transcripts). STARmap PLUS further advances the technology, detecting a greater number of targeted genes together with biomarker proteins.

## 3 smFISH (Barcoded) Based Technologies

### 3.1 MERFISH

**Figure 2:**
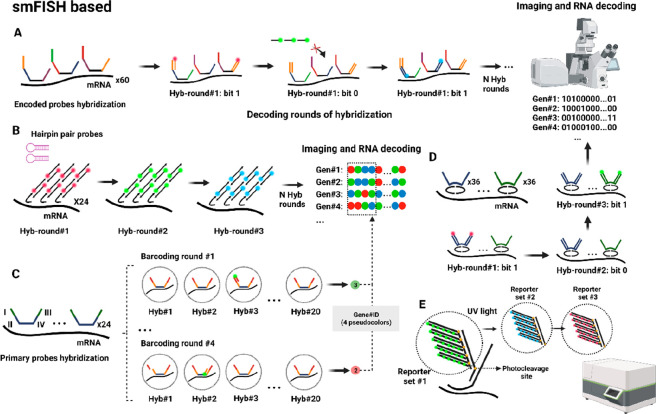
smFISH-based technologies. (A) MERFISH encodes each mRNA molecule with a unique *N*-bit word, which are decoded by hybridization with encoded probes with flanked regions recognized by fluorescently labeled probes in *N* rounds of hybridization that assign bit-1 (detection) or bit-0 (no detection). (B) In HCR-seqFISH, each mRNA molecule is hybridized with fluorescent hairpin probes that generate a fluorescent self-assembly polymer, which is imaged and stripped, followed by a new round of hybridization. (C) SeqFISH+ assigns a four-pseudocolor code to each mRNA molecule by separating three channels in 60 pseudocolors. (D) Split-FISH uses split probes to hybridize to the target RNA and then decodes each mRNA molecule as an *N*-binary word through encoded and detection probes. (E) In Nanostring SMI, a fluorescent code is generated for each mRNA molecule using a tree amplification and a combination of fluorescently labeled reporters that after imaging are cleaved with UV light.

In 2015, Zhuang’s lab published multiplexed error robust FISH (MERFISH),^41^ assigning each gene an *N*-bit binary code word detected through rounds of hybridization with encoding and readout probes. The barcoding strategy begins with fixing cells or fresh frozen tissues, followed by the initial hybridization of encoding probes to mRNA targets, each encoding probe with two flanked readout sequences on each end. A total of 192 encoded probes are used for one mRNA, divided into two groups: 96 probes are flanked by readout sequences I and II, and the other 96 are flanked by sequences III and IV. Then, fluorescently labeled readout probes are employed to identify the readout sequences, generating signals during each hybridization round. Among the hybridization rounds, the readout probes are cleaved. The presence of signal is designated as “bit-1”, while the absence of signal is designated as “bit-0”. Through *N*-rounds of hybridization, the *N*-bit binary code is decoded for each mRNA species (Figure 2A). An error encoding scheme called the Modified Hamming Distance of 4 (MHD4) was introduced. In this scheme, the “bit-1” remains constant at four in the *N*-bit binary code for each gene, while the rest are set as “bit-0”. This approach ensures that mishybridization in one round does not impact the detection of a specific mRNA.

MERFISH detects 140 targeted RNA species using a 16-bit MDH4 code and 1001 targeted RNA species without a correction scheme using a 14-bit HD2 in human fibroblast cells,^41^ with a efficiency of 80% to 95%.^41,42^ It offers high multiplexing capacity and good correlation with bulk RNA-sequencing data, used in molecular architecture and disease studies.^43,44^ In 2018, MERFISH combined with expansion microscopy detected ∼10,000 transcripts using a 69-bit HD4 encoding scheme with 80% efficiency in fixed cells and enhanced SBR ratios.^42^ The method has limitations such as specialized equipment and high costs due to the requirement for numerous probes. This high number of probes also leads to lower SBR ratios. However, the technology has been automated as MER-Scope, enabling end-to-end analyses and making it accessible and compatible for formalin-fixed paraffin-embedded (FFPE) samples.

### 3.2 SeqFISH/HCR-seqFISH

In 2014, the Cai lab developed seqFISH, combining combinatorial transcript detection and super-resolution microscopy.^45^ In seq-FISH, the barcoding strategy assigns color-codes to each mRNA species. This is done by using a set of fluorescent probes that hybridize along the mRNA, followed by imaging and clearing the probes. Then, a new hybridization round is initiated with a second set of probes with a distinct fluorophore. After several hybridization rounds, this process generates a unique color-code associated with a specific mRNA species. The method was employed for the detection of 12 mRNAs in yeast cells using 4 fluorophores and 5 hybridization rounds. In 2016, HCR-seqFISH combined seqFISH with single-molecule hybridization chain reaction (smHCR) in a similar barcoding strategy in hydrogel-embedded tissues,^46^ improving the efficiency and multiplexing capacity. In this case, hairpin probes are used to initiate HCR amplification, generating fluorescent polymers along the mRNA. Then, DNase removes the amplification polymers, allowing for a second round of hybridization using the same primer set but with a different fluorophore. After *N* rounds, unique color-codes are generated for each mRNA. An additional round of hybridization was included as an error correction scheme to account for any possible signal loss (Figure 2B).

HCR-SeqFISH detected 249 targeted genes in 16,958 single cells in 25 μm mouse hippocampus sections with 84% efficiency.^47^ While HCR-seqFISH offers robust RNA detection and moderate multiplexing, it also experiences challenges such as lower SBR and the necessity for super-resolution microscopy, limiting the widespread implementation.

### 3.3 SeqFISH+

In 2019 the same group published seqFISH+, enhancing targeted multiplexing by separating 60 pseudocolors across three fluorescent channels in confocal microscopy.^48^ SeqFISH+ uses primary probes targeting mRNA in fixed fibroblast cells with a central region for mRNA targeting and two overhang sequences having four encoded regions (I–IV) with unique gene-specific barcode sequences. Fluorescently labeled readout probes read these sequences, resulting in a four-signal code for each targeted RNA species. The SeqFISH+ process begins with 24 primary probes per mRNA molecule added to the fixed sample. After hybridization, primary probes are cross-linked with an embedded hydrogel (Figure 2C). The group detected 10,000 RNA species, averaging 3,333 transcripts per channel through four barcoding-rounds of hybridization, each divided into 20 pseudocolor readout-rounds, where each transcript is sampled once with a fluorescent probe. Over 80 hybridization rounds, four-signal codes are generated per transcript, detecting 3,333 transcripts per channel. An extra hybridization round was introduced for error correction.

SeqFISH+ exhibited a 49% multiplexing efficiency,^48^ detecting 10,000 targeted genes in fibroblast cells simultaneously. The group also profiled 10,000 transcripts in 5 μm sections of mouse brain, covering a 0.5 mm^2^ area. Despite using confocal microscopy and avoiding super-resolution equipment, the large number of probes makes the technology challenging to spread. Automating the process could help to expand this technique to other research groups.

### 3.4 Split-FISH

In 2020, Chen’s group developed split-FISH,^49^ a method using two split probes targeting mRNA (Figure 2D). Only when both split probes hybridize adjacent to each other is the needed complementary base pairing present to enable the hybridization of bridge probes. Bridge probes contain a central region that recognizes the split barcode and two identical flanked readout sequences detected by fluorescent-labeled probes. With this process, the group avoided off-target nonspecific binding of probes and enhanced the SBR ratio. The group used a 26-bit scheme to create a 317-combinatorial library with two constant “bit 1” for each mRNA species and remaining bits set to “0”. They divided 72 pairs of split probes into two pools, each presenting a unique barcode recognized by a bridge probe. After 13 hybridization cycles, the 26 bits were decoded, enabling the detection of 317 mRNA species in 7 μm sections of fresh frozen mouse brain and liver tissues. Eight nonspecific codewords were included as controls for false negatives.

Split-FISH has ∼71% efficiency and can detect multiplexed transcripts in unclear tissues with better SBR, but its moderate multiplexing capacity is limited by using only one barcoded bridge.

### 3.5 Nanostring Spatial Molecular Imaging (SMI)

Nanostring’s spatial molecular imaging (SMI) technology, published in 2022, measures targeted RNAs and proteins in FFPE and fresh frozen tissues.^50^ SMI chemistry involves five barcoded oligos per gen to create a branched amplification, each barcode oligo with a gene-specific target-domain and a shared readout-domain. The readout domain is divided into four individual barcodes detected sequentially by recognition probes with multiple sites for fluorescently labeled reporters. The recognition probe and reporters are attached to UV-photocleavable sites. After imaging, the UV-light activates the photocleavable sites, removing these probes to proceed with the next rounds of hybridization (Figure 2E).

The 64-bit HD4 encoding scheme enabled detection of 980 RNAs and 108 proteins in 5 μm sections of lung and breast cancer FFPE tissues. An average of 260 transcripts were detected per cell among 769,114 analyzed cells. SMI also allows co-detection of transcripts and proteins in large scan areas (16–375 mm^2^). While SMI enables robust targeted gene expression analysis, limited transcripts and proteins per cell detection may constrain transcriptional studies. However, Nanostring offers automated SMI equipment, increasing accessibility.

In summary, barcoded smFISH-based technologies show high efficiency and multiplexing capacity. However, many of these methods have lower SBR ratios due to the need for numerous probes, and also several of them need specialized equipment. MERFISH provides excellent multiplexing capacity and has been automated as a MER-scope, making it more widely accessible. HCR-seqFISH exhibits robust efficiency with moderate multiplexing capacity but requires super-resolution microscopy. SeqFISH+ offers great multiplexing capacity and efficiency using confocal microscopy, although the extensive number of probes used makes it challenging to adopt without automation. Split-FISH allows multiplexed transcript detection in unclear tissues with improved SBR but has a moderate multiplexing capacity. SMI-Nanostring enables moderate multiplexing of RNA and protein measurements in FFPE and fresh frozen tissues in an automated format.

### 3.6 ISS and smFISH Comparison

ISS and smFISH methods offer exciting opportunities for transcriptomics analyses, employing diverse approaches and barcoding strategies, each with its own advantages and disadvantages. In ISS, the barcoding strategy involves recognizing cDNA or mRNA using barcoded padlock probes (ISS) or circularizing the cDNA (FISSEQ), performing RCA, and decoding the amplicons with SBL (ISS), SBH (HybISS, HybRISS), or modified versions, such as SEDAL (STARmap). On the other hand, in barcoded smFISH, encoded probes initially hybridize to the RNA and are then decoded in subsequent hybridization rounds, assigning an *N*-bit code word (MERFISH, Split-FISH, SMI-Nanostring) or color-codes to each mRNA species (HCR-seqFISH, seqFISH+).

In ISS, target amplification allows for higher SBR ratios using a smaller number of probes compared to the smFISH methods. However, the size of the amplicons can lead to imaging overcrowding, limiting the multiplexing capacity to hundreds of targets per cell. To overcome this limitation, strategies such as hydrogel-embedding in STARmap and expansion microscopy in ExSeq have been employed, increasing the multiplexing capacity to thousands of targets. On the other hand, the barcoding scheme used in smFISH-based methods enables a high multiplexing capacity, allowing the detection of thousands of gene transcripts with high efficiency. However, the use of a large number of probes also leads to a low SBR ratio due to nonspecific binding of the probes to cellular components such as proteins and lipids.^42^ Various strategies have been implemented to address this issue. For instance, in MERFISH and seqFISH+, the RNA content is anchored to a polymer matrix, followed by sample clearing to remove proteins and lipids, thereby improving the SBR ratio. In split-FISH, mRNA is detected only when split probes hybridize consecutively, reducing off-target of probes and resulting in a higher SBR. Additionally, other approaches like enhanced electric FISH (EEL-FISH)^51^ involve the electrophoretic transfer of the RNA content to a capture surface, effectively clearing the sample and avoiding nonspecific binding to other cellular components. A representation of all technologies along with their respective publication years is found in Figure S1. A detailed comparison between ISS and smFISH-based methods is presented in Table S1.

## 4 Spatial Capturing Technologies

### 4.1 ST–10x Visium

**Figure 3:**
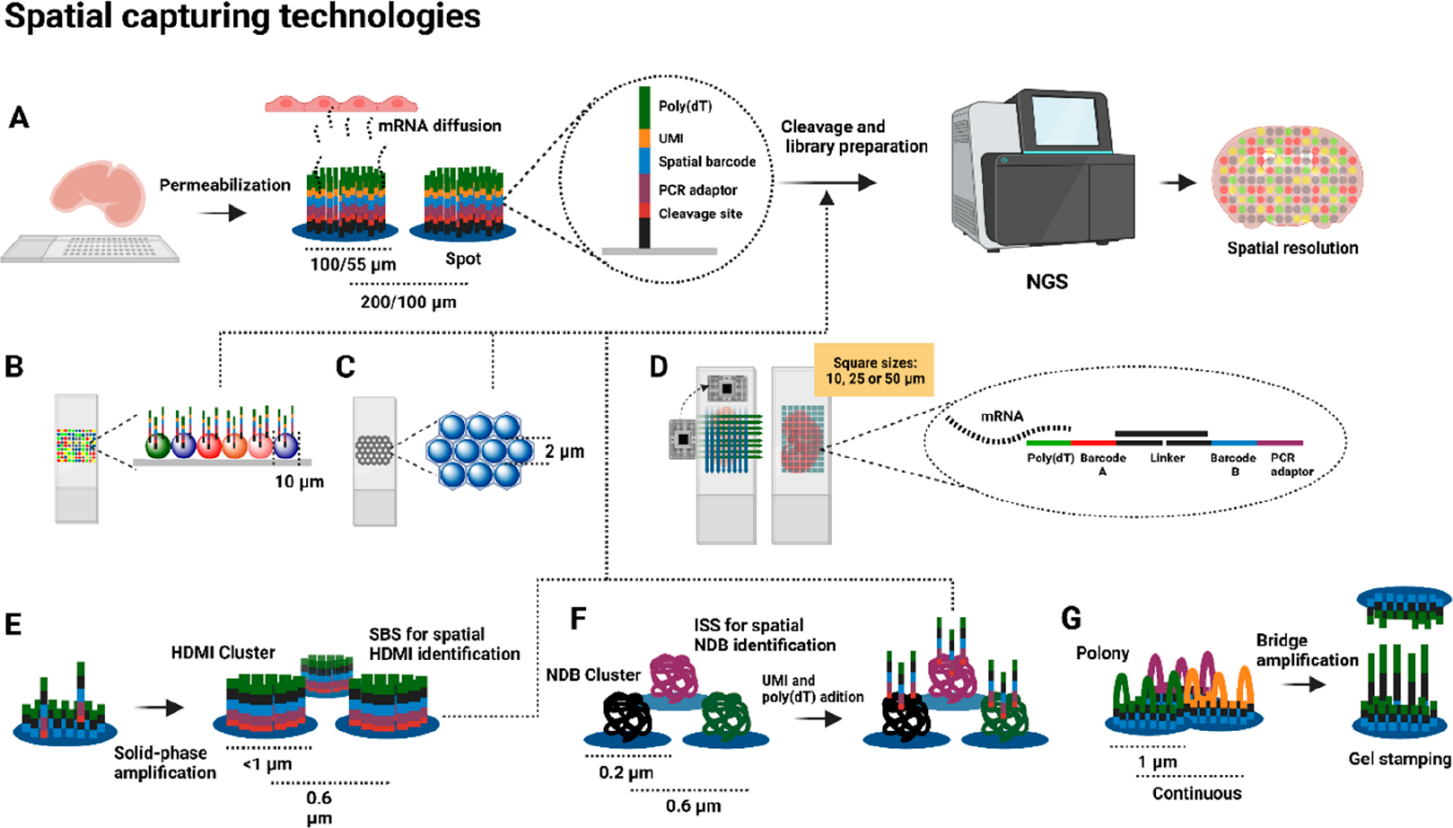
Spatial capturing technologies. (A) In ST/Visium, tissues are placed on oligo arrayed slices with spots of 100–55 μm. After permeabilization, mRNA diffuses to the oligo(dT) for cDNA synthesis, cleaved for library preparation, and NGS, enabling spatial resolution of gene expression in tissues. (B) In Slide-seq, the capturing area is formed by 10 μm beads, while (C) HDST uses beads of 2 μm placed in hexagonal walls. (D) In DBIT-seq, two microfluidic chips generate perpendicular flows generating squares of 10, 25, or 50 μm for mRNA capturing and cDNA synthesis. (E) Seq-Scope creates HDMI clusters of a diameter less than 1 μm on the surface through PCR amplification. (F) Stereo-seq generates the pattern using DNA nanoball clusters of 0.2 μm diameter, while (G) Pixel-seq generates a continuous array using PCR amplification, with the possibility to reproduce the array using gel stamping.

In 2016, Lundeberg’s lab developed spatial transcriptomics (ST), a widely used commercial method for transcriptome-wide analyses.^1^ This technology covers transcriptome-wide analyses by capturing the RNA content before sequencing. ST is based on attaching spatially barcoded oligos in 100 μm spots on glass slides with a spot–spot distance of 200 μm. The oligos contain a cleavage site, PCR handling sequence, spatial barcode, UMI sequence, and oligo(dT) domain. Once the arrayed slides were generated, tissues are placed on top and are fixed, permeabilized, stained, and imaged to visualize the tissue morphology. During the permeabilization step, mRNA molecules diffuse vertically from the tissue and are captured by the oligo(dT) domain. Then, RT is conducted, followed by tissue digestion and cleavage of the oligos from the array. The cDNA collected is processed for library preparation and Illumina sequencing (Figure 3A). The spatial barcode on the primers is related to its localized 100 μm spot, which covers 10–50 cells, resulting in transcriptomes that are spatially resolved at a 100 μm resolution.

ST, the first spatial capturing technology, enables transcriptome-wide and *de novo* analyses. In Alzheimer’s disease research, ST detected 31,283 ± 7,441 UMIs and 6,578 ± 987 unique genes in 100 × 100 μm^2^ mouse brain tissue areas.^17^ The technology, now acquired by 10x Visium, has a 55 μm spot diameter and 100 μm spot-to-spot center and covers areas up to 42.25 mm^2^. Adapted for both FFPE and fresh frozen tissues, Visium also enables co-detection of proteins.^52^ ST has been combined with other methods, such as Spatial Multi-Omics (SM-Omics),^53^ and expanded for whole transcriptomics analysis.^54^ While ST is widely used in biological studies,^55,56^ its main limitation is the lack of single-cell resolution.

### 4.2 Slide-seq/Slide-seqV2

In 2019, the Macosko lab developed Slide-seq, reducing the spot-size to 10 μm using barcoded microparticles on slides with a 10 μm spot-to-spot distance to enable high-resolution spatial transcriptomics.^57^ The process followed is similar to ST, with barcoded oligonucleotides attached to the microparticles. The tissue is placed on slides with these microbeads pooled, fixed, permeabilized, stained, and imaged (Figure 3B). The group later improved the technology with Slide-seqV2 in 2021, enhancing capture efficiency and introducing novel array generation and library preparation strategies.^56^ Slide-seqV2 showed increased capture efficiency, detecting 550 UMIs per microparticle and 45,772 UMIs per 10 × 10 μm^2^ areas in mouse hippocampus fresh frozen tissues, covering a 7 mm^2^ area.^58^ While Slide-seq enables near single-cell resolution and *de novo* transcriptome-wide analyses, its limitation lies in low transcript detection, requiring the grouping of areas. In addition, 30% of analyzed microparticles may capture transcripts from multiple cells, potentially affecting single-cell resolution.^58^

### 4.3 HDST

In 2019, high-definition spatial transcriptomics (HDST) technology^59^ employed smaller 2 μm barcode microparticles with a 2 μm spot-to-spot distance, covering 13.68 mm^2^ areas. Microparticles are placed in hexagonal wells and attached to barcoded oligos. To decode each microparticle’s location, multiple hybridization rounds with fluorescently labeled oligonucleotides are conducted. Mouse brain tissue sections are placed on the microparticle array, fixed, permeabilized for mRNA diffusion, stained, and imaged by microscopy. After cDNA synthesis, it is digested for barcode transcript extraction, library preparation, and Illumina sequencing (Figure 3C). Capture efficiency in mouse brain cryosections was 7.1 ± 6.0 UMIs per microparticle and 11.5 UMIs per 10 × 10 μm^2^ area. Binning several microparticles, like grouping 5 hexagonal wells, yielded 44.4 ± 30.6 UMIs. Despite HDST’s 2 μm spot diameters for near single-cell resolution, the low number of captured transcripts necessitated spot grouping for analysis. This approach, spanning multiple cells, may challenge the accurate identification of cell-specific gene expression patterns.

### 4.4 DBIT-seq

Rong Fan’s group developed deterministic barcoding in tissue (DBIT-seq) in 2020 for detecting mRNAs and proteins in FFPE and frozen tissue sections.^60^ This technique uses a polydimethylsiloxane (PDMS) microfluidic chip with 50 parallel microchannels, placed on the tissue with each channel supplied with a different barcoded oligo A solution (containing a spatial barcode, a ligation linker, and an oligo(dT) domain). When solution A passes through the tissue, mRNA is captured by the oligo(dT) domain and RT takes place. Then, a second PDMS chip with perpendicular channels is introduced, containing barcoded oligos B (containing a second set of barcoded oligos B, with a ligation linker, an UMI sequence, and a PCR adaptor). T4 ligase facilitates the ligation of oligos A and B to create a unique combination when they overlap. For instance, if the overlap is in file 12 and row 34, the resulting region will be spatially identified as A_12_B_34_. The spatially barcoded mosaic is generated, the tissue digested, and cDNA collected for library preparation and NGS (Figure 3D).

The square size can be 10 × 10 μm^2^ or 50 × 50 μm^2^, and the total capture area is 1 mm^2^ or 25 mm^2^, respectively. In 10 × 10 μm^2^ square areas on fixed mouse embryo tissue sections, they detected an average of ∼5,000 UMIs and 2,068 genes in 1 mm^2^. In whole mouse embryo sections at 50 × 50 μm^2^ square areas, they detected 12,314 UMIs and 4,170 genes per square. With protein colocalization, they detected 3,038 UMIs and 22 proteins per 50-square. DBIT-seq improved UMI capture efficiency compared to Slide-SeqV2 and HDST, utilizing an easy-to-implement microfluidic device. However, limited channels and empty spaces still hinder single-cell resolution.

### 4.5 Seq-Scope

In 2021, Lee’s lab introduced Seq-Scope, an adaptation of the Illumina sequencing platform,^61^ achieving a submicrometric spot-to-spot distance of 0.6 μm in a 0.2 mm^2^ capture area. Seq-Scope involves two sequencing rounds: creating a spatial array and capturing cDNA information. First, oligonucleotides are attached to a surface with multiple sequences, forming a high-definition map coordinate identifier (HDMI) cluster array. Sequencing-by-synthesis (SBS) is then used to identify and locate each HDMI cluster. In the second round, tissue is placed on the HDMI array, the tissue is permeabilized, mRNA is captured, RT is carried out, and the cDNA is collected for library preparation and NGS (Figure 3E).

Seq-Scope attained impressive capture efficiency in mouse fresh frozen tissues: 6.70 ± 5.11 UMIs (liver), 23.4 ± 17.4 UMIs (colon), 5.88 ± 4.22 (liver), and 19.7 ± 14.3 (colon) genes per HDMI cluster. In colon tissues, grouped areas of 10 × 10 μm^2^ detected an average of 2743 UMIs. When grouped into single-cell areas, the output was 4,734 ± 2,480 UMIs and 1,673 ± 631.7 genes per cell. Seq-Scope achieved transcriptomics outputs with remarkable submicrometer spatial resolution. However, limitations include cost, time in generating the HDMI array, restriction to poly(A) domains, and the inability to introduce co-detection of proteins in tissue analyses.

### 4.6 Stereoseq

In 2022, BGI Research’s team developed Stereo-seq using a modified DNA nanoballs (DNB) based sequencing approach.^62^ DNBs are created via RCA and deposited onto an array using a MGI DNBSEQ-Tx sequencer, resulting in 400 spots per 100 μm^2^ area.^62^ The array undergoes ISS to obtain the spatial coordinate identity (CID) from each DNB. UMI and oligo(dT) are hybridized onto DNB spots, and embryonic mouse frozen tissues are placed on the array. After permeabilization, mRNA diffuses to the array, and cDNA is collected for library preparation and NGS (Figure 3F).

Capture efficiency ranges from an average of 69 UMIs per 2 μm diameter (3 × 3 DNBs) to 133,776 UMIs per 100 μm area (140 × 140 DNBs). Total tissue capturing area can be 50, 100, or 200 mm^2^. Analyzing 50 × 50 DNB sections of embryonic frozen tissues, Stereo-seq detects 1,770 to 3,900 genes and 4,357 to 13,789 UMIs. Its high capture efficiency and submicrometer resolution allow for larger capture areas compared to other technologies. Limitations include cost and time for creating DNB arrays, compromised single-cell resolution due to region grouping, and the potential misrepresentation of low-expression transcripts due to small spots.

### 4.7 PIXEL-seq

Polony-indexed library sequencing (PIXEL-seq) was introduced by Liangcai Gu’s lab in 2022.^63,64^ Like Seq-scope, it relies on generating PCR or DNA cluster arrays called polonies. The method involves attaching spatially barcoded oligos with poly(dT) domains and restriction sites to a polyacrylamide (PAA) gel. DNA bridge amplification creates polonies on the PAA gel, followed by *Taq*I digestion, exposing the poly(dT) domain. The polonies underwent SBS to generate a spatial index map. Mouse brain frozen sections are placed onto the gel for mRNA capture and cDNA synthesis, followed by UMI introduction and NGS library preparation (Figure 3G).

Polonies range from 1.07 to 0.906 μm in size and are continuously distributed on the array. Analyses can be conducted at 2 × 2 μm^2^, 10 × 10 μm^2^, or 50 × 50 μm^2^ tissue area resolution, capturing ∼1000 UMIs in 10 × 10 μm^2^ areas. In a 13 mm^2^ section, 82.5 million UMIs (1–678 UMIs per barcode) were detected. PIXEL-seq’s primary advantage is minimal space between polony clusters. The method was also developed as a scalable stamping technique, incorporating an automated device for gel-to-gel DNA copying, reducing costs and time. The main limitation is the analysis of grouped clusters, with PIXEL-seq showing less cell type separation compared to the dissociative scRNA-seq of brain tissues.

In summary, spatial capturing technologies enable transcriptome-wide analyses by capturing the RNA content before sequencing, in contrast with ISS and smFISH methods, which are typically limited to preselected targets. Among these technologies, ST/Visium is the commercial option more available, offering *de novo* analysis with a resolution of 100 μm, but it falls short of achieving single-cell resolution. To address this limitation, subsequent technologies have made advancements toward reducing the size of arrayed areas. Slide-seq and Slide-seqV2 use barcoded microparticles of 10 μm on slides, while HDST offers smaller spot diameters of 2 μm, approaching single-cell resolution. DBIT-seq improves the capture efficiency through an easy-to-implement microfluidic device. Seq-scope and Stereo-seq achieve submicrometric spatial resolution, and Pixel-seq was developed as a continuous distribution of polonies and a scalable stamping method.

However, a challenge with these improvements is that reducing the capture area can lead to a decreased capture efficiency. As a result, most transcriptomic analyses require grouping of multiple areas, potentially affecting accuracy and hindering the achievement of single-cell resolution. Currently, a key focus in advancing these technologies is finding a balance between reducing the detection area and maintaining an improved capture efficiency. A comprehensive comparison of the spatial capturing technologies can be found in Table S2.

## 5 Light-Based ROI Selection Technologies

### 5.1 Nanostring the GeoMx Digital Spatial Profiling (DSP)

**Figure 4:**
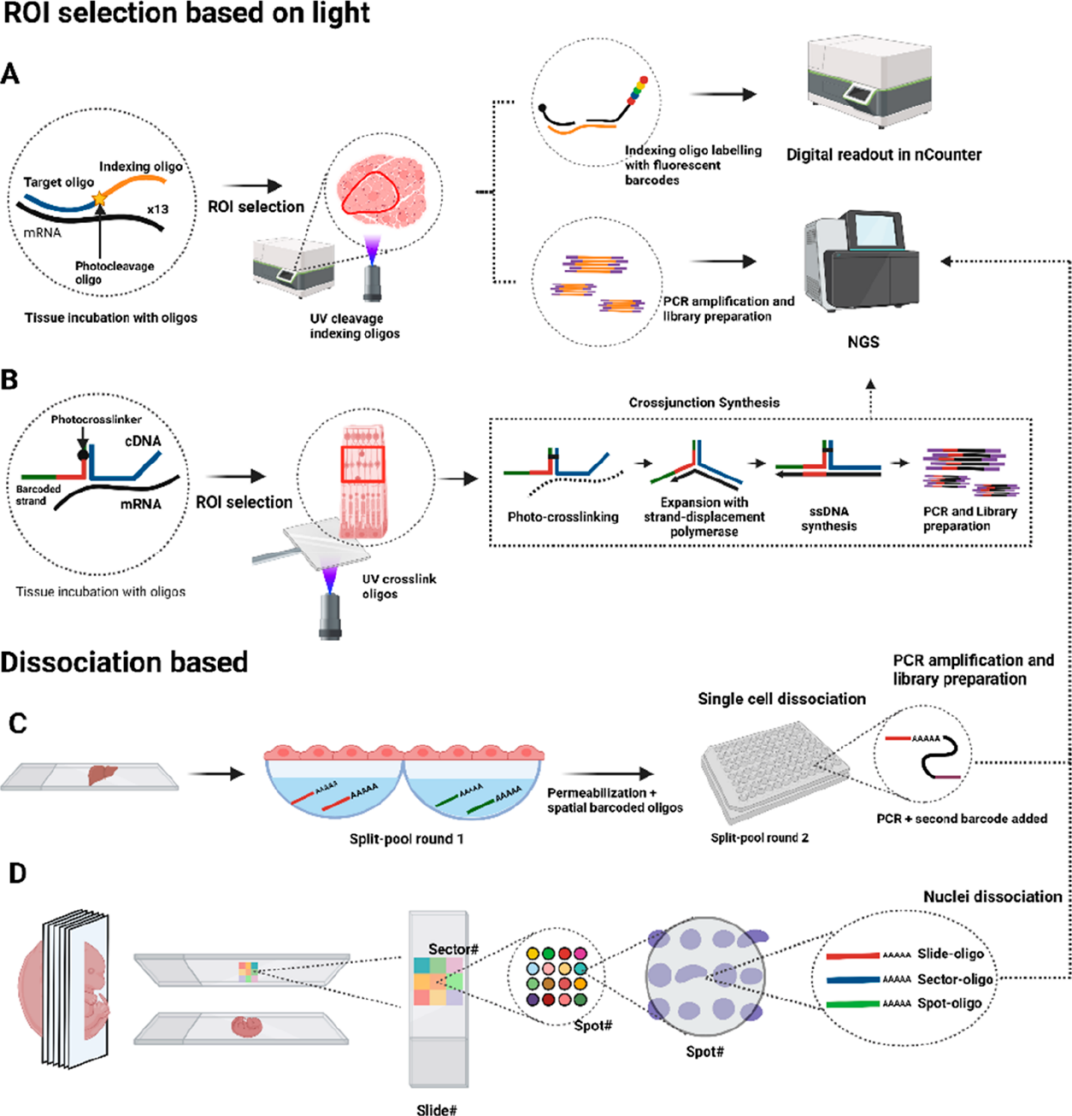
Light-based ROI selection and spatial single-cell/nuclei dissociation technologies. (A) Nanostring DSP uses oligos with a target domain and an indexing oligo separated by a photocleavage site. (B) Light-Seq hybridizes cDNA with a barcoded oligo that carries a photo-cross-linker for a crossjunction synthesis. (C) In XYZeq, tissues are incubated on walls with oligo(dA) and permeabilization buffer, and barcoded cells are separated in a second split-pool round followed by scRNA-seq. (D) Sci-Space uses an arrayed slide with hashing oligos to spatially barcode a group of nuclei. After barcoding, the sample is digested and prepared for sciRNA-seq.

Nanostring introduced GeoMx digital spatial profiling (DSP) in 2019 for detecting RNA species and proteins in FFPE and fresh frozen tissues.^65^ DSP uses oligonucleotides with two sequences separated by a UV-photocleavage linker: an RNA target domain and a fluorescently labeled indexing domain as an mRNA barcode. After tissue characterization, digital images are scanned to select ROIs with a digital mirror device. UV-light releases indexing oligos in ROIs, which are collected and identified by using nCounter equipment or NGS analyses (Figure 4A). For protein co-detection, indexing oligos are conjugated to primary antibodies.

In a study with 400 μm diameter ROIs on FFPE human colorectal samples, 96 transcripts (928 RNA probes) and 44 proteins were profiled using nCounter, while 1,412 genes (4,998 RNA probes) were profiled using NGS.^63^ Nanostring DSP’s automation allows for wider adoption and protein co-detection. However, the method’s main limitation is the labor-intensive manual selection of a limited number of ROIs, hindering whole tissue transcriptomics analyses.

### 5.2 Light-seq

Yin’s lab introduced Light-seq in 2022, using ultrafast cross-linking chemistry of barcoded oligos to RNA species with UV-light.^66^ The process begins with RT of mRNAs in fixed mouse retinal cryosections using random primers with a 5′ barcoded overhang. Then, cDNA molecules are polyadenylated at their 3′ end, and the sample is incubated with barcoded sequences containing photo-cross-linker 3-cyanovinylcarbazole nucleoside (CNVK) and a UMI sequence. ROIs are selected, and a custom 2 μm resolution photomask illuminates them with UV, cross-linking CNVK-UMI sequences to cDNA. This results in the formation of covalent bonds between the cDNA and the CNVK-barcode oligo. Next, the barcoded cDNAs are extracted using RNase H. Then, a cross-junction synthesis reaction (similar to that used in SABER-FISH) copies the cDNA sequence and the barcode to a single-stranded DNA for PCR amplification and NGS (Figure 4B). RNase-H degrades only RNA in the RNA–DNA complexes, meaning the rest of the tissue sample is not affected and can be imaged multiple times. Light-seq sensitivity is low (∼4%) compared to single-molecule FISH, but UMI detection efficiency is comparable to spatial capturing methods (∼1,000–10,000 per 10 × 10 μm^2^ area). Similar to Nanostring DSP, ROI selection can be laborious for whole tissue section analysis.

## 6 Spatial Cell/Nuclei Dissociation Technologies

### 6.1 XYZeq

XYZeq, developed by the Ye lab in 2021,^67^ utilizes two rounds of split and pool barcoding to generate single cells as spatially barcoded spots for sequencing. Based on single-cell combinatorial indexing (sci) RNA-seq, XYZeq allows for the analysis of single cell pools from multiple samples in one experiment. Initially, mouse liver and tumor fresh frozen sections are fixed and placed on arrays with 500 μm diameter microwells containing spatially barcoded RT primers and a dissociation/permeabilization buffer. The tissue is permeabilized, allowing barcoded oligos to diffuse to the wells. After RT, spatial barcodes and UMI sequences are introduced, assigning cells to specific wells. Cells are then pooled in new wells for a second round of indexing through RT and PCR, introducing a second barcode. Each cell acquires a combinatorial barcode (spatial location, UMI, and PCR handling) for pooling and sci-RNA-sequencing (Figure 4C). XYZeq generates 294,912 single-cell barcodes, detecting ∼1000 UMIs and 300–600 genes per mouse cell using 25 μm tissue sections of mouse frozen liver or tumor. The main limitation is a spatial resolution limited to the 500 μm diameter microwells, making it infeasible to achieve single-cell resolution.

### 6.2 Sci-space

In 2021, the Trapnell group introduced spatial resolution to their Sci-Plex technology, creating Sci-space.^68^ This method uses indexed slides with unique combinations of barcoded oligos (hashing oligos) deposited onto spots in dried agarose-coated slides. With a 73.2 μm spot-diameter and 222 μm spot-to-spot distance, hashing oligos comprise slide, sector, and spot oligos with a poly(dA) domain to determine each nucleus’s spatial location. The poly(dA) domain labels nuclei for identification during sci-RNA-seq experiments. The spatial position of each spot is determined using a unique identifier from the slide, sector, and spot oligo combination. A 14-day mouse embryo section is placed on the patterned array and permeabilized with a specific slide oligo, forming a sandwich between the slide and the tissue section. Hashing oligos transfer from the spots to the nuclei in the tissue, which is then imaged, and nuclei are dissociated for sci-RNA-seq (Figure 4D).

Each slide contains 7,056 uniquely barcoded spots spanning an 18 mm^2^ area. After sci-RNA-seq, an average of 2,514 UMIs and 1,231 genes are detected per cell, identifying 164 nuclei/mm^2^ or 2.2% of the estimated nuclei in the sample. Sci-space’s resolution is limited by the patterned array of hashing oligos (200 μm), and the transcriptional analyses include only transcripts from nuclei.

In summary, methods that select ROIs via UV-light stand by the possibility to study specific tissue areas and rare cell types in resolved areas with a capturing efficiency similar to spatial capturing technologies, with the advantage of Nanostring DSP as an automated system. The main limitation of ROI-methods is the impossibility of studying larger or whole tissue sections as the selection of multiple ROIs can be laborious. Methods based on the dissociation of single cells or nuclei as barcoded spots are a different concept with a natural approach for single-cell studies; however, the spatial resolution is limited by the patterned array used to create the spots for barcoding, although with capturing efficiencies per cell similar to those achieved by spatial capturing methods. A detailed comparison of light-based ROIs and dissociation-based technologies is presented in Table S2.

## 7 Data Analysis

Spatial RNA methods require several processing steps and generate large amounts of data.^10^ Complete pipelines like *Starfish* for ISS and smFISH and *Space Rangers* for ST/Visium have been developed for data analysis. Cell segmentation, aided by machine learning toolkits such as *Ilastik*, is crucial for generating gene expression matrices in ISS and smFISH-based techniques. Capturing-based methods require deconvolution techniques such as negative binomial models and non-negative matrix factorization, among others.

Normalization, like transcript per million (TPM) ratio or using packages such as *Scanpy*, is needed before clustering to identify expression patterns using methods like PCA.^67^ Gene scoring assigns expression measures to genes, and scRNA-seq data can guide the spatial transcriptomic experiment design and interpretation. Deep learning methods like *GimVI* or *Tangram* and *Python* packages like *Seurat* or *scVI* integrate scRNA-seq data with spatial data.^69−71^

*De novo* transcriptomics assembles and annotates transcripts using reference databases or gene prediction algorithms. Spatial transcriptomics data can be used to decipher various biological processes, such as spatially variable genes using Gaussian process regression (GPR), cell–cell interactions (e.g., *MISTy*, *stLearn*), gene–gene interactions (SpaOTsc, MESSI), ligand–receptor pair detection (e.g., *CellPhoneD*), or predicting gene expression levels based on histology images (*PathoMCH*).^2,72^

## 8 Conclusions

Spatial transcriptomics techniques have significantly expanded in recent years, enabling researchers to analyze gene expression patterns at the individual cell or tissue levels in various fields. ST/Visium, Nanostring DSP, LCM, and Tomo-seq are widely used, with MERFISH and ISS^2^ being popular for targeted studies. ISS and smFISH-based methods allow for single-cell studies of preselected genes, while capturing and dissociation methods enable transcriptome-wide and *de novo* studies, although with lower efficiency and resolution.^1^

Most methods have been developed for fresh frozen human or mouse brain tissues, but Nanostring DSP, ST/Visium, LCM, DBIT-seq, and the commercialized version of MERFISH (MER-scope) are also suitable for FFPE tissues. Some methods, like Nanostring SMI, STARmap PLUS, ST/Visium, DBIT-seq, and Nanostring DSP, have been developed for protein co-detection. When selecting a spatial transcriptomics method, it is crucial to prioritize its effectiveness in addressing the research question and consider its accessibility for implementation. Integrating multiple spatial methods^17^ or combining spatial data with scRNA-seq data^73^ has shown potential in deciphering RNA function in its spatial context.

## 9 Future Perspective

The field of spatial transcriptomics is constantly evolving, and one promising area is the integration of spatial transcriptomic data with other spatial omics technologies, such as proteomics, epigenomics, or metabolomics. To promote the broader adoption of spatial transcriptomics techniques in the research community with the aim to eventually reach clinical applications, it is necessary to focus on reducing the costs and developing automated formats for data acquisition and analysis. While the core technologies are ready, there is still the need to invest in developing cost-effective automated platforms and the creation of user-friendly software tools. In order to avoid biases, the scientific community must provide open data sets of the tissues analyzed for comparison, as well as precise data on the detection or capture efficiency of novel methods. By providing curated data, the field of spatial transcriptomics will quickly benefit from artificial-intelligence-based large language models. Consequently, it is the responsibility of the spatial transcriptomics community to provide this curated data in order to fully maximize the potential of such advanced AI systems.

While there is no perfect method, there is still room for improvement in enhancing the detection or capture efficiency toward single-cell resolution. In the meantime, the integration of various spatial transcriptomic methods or the guidance of scRNA-seq data may be necessary for some biological studies. In addition, despite some studies including noncoding RNAs,^38,54^ most of them do not or are not currently able to detect small RNAs such as microRNAs. Therefore, incorporating the detection of these noncoding RNAs as well as developing techniques compatible with small RNAs will be critical for obtaining a comprehensive understanding of the spatial gene expression and regulation.

We anticipate that spatial transcriptomics will have a significant impact not only in the fields of cancer genomics and biomarker discovery but also in drug development. At present, RNAs can be regarded as golden biomolecules as they can serve as biomarkers while also possessing a unique duality: they can be both drugs and small molecule druggable targets. This implies that cost-effective technologies capable of spatially locating RNAs and their mimics will be key enabling technologies for this new RNA-based era of medicine.

## Supporting Information Available

Spatial transcriptomics timeline (Figure S1); ISS and barcoded smFISH comparison (Table S1); spatial capturing, microdissection, light-based ROIs, and dissociation technology comparison (Table S2) (PDF)
