# Supplementary material for: Spatial Transcriptomics: Emerging Technologies in Tissue Gene Expression Profiling
Source: Anal Chem. 2023 Oct 10;95(42):15450–60. doi: 10.1021/acs.analchem.3c02029 (PMC10603609; doi:10.1021/acs.analchem.3c02029)
Supplement: Supplementary file 1 — ac3c02029_si_001.pdf [file ac3c02029_si_001.pdf]

Supporting Information

Spatial Transcriptomics: Emerging Technologies in Tissue Gene Expression Profiling

Agustín Robles-Remacho*[1,2,3], Rosario M. Sánchez-Martín*[1,2,3], Juan José Díaz-Mochón*[1,2,3]

[1]GENYO. Centre for Genomics and Oncological Research: Pfizer / University of Granada / Andalusian Regional Government. PTS Granada. Avenida de la Ilustracion, 114. 18016 Granada, Spain.

[2]Department of Medicinal and Organic Chemistry, School of Pharmacy, University of Granada, Campus Cartuja s/n, 18071 Granada, Spain.

[3]Biosanitary Research Institute of Granada (ibs.GRANADA), University Hospital of Granada/University of Granada, Avenida del Conocimiento, s/n, 18016 Granada, Spain.

*Corresponding authors: Agustín Robles-Remacho (agustin.robles@genyo.es), Rosario M. Sanchez-Martin (rosario.sanchez@genyo.es) and Juan José Díaz-Mochón (juanjose.diaz@genyo.es)

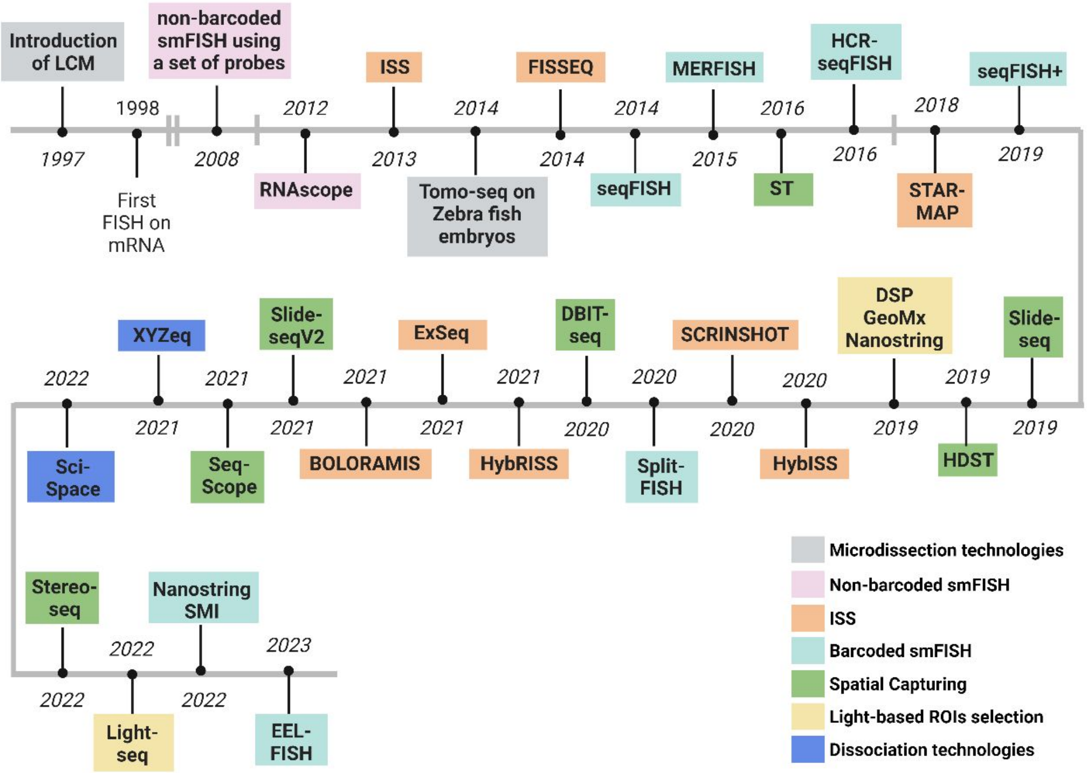

Figure S1. Spatial Transcriptomics Timeline. Scheme of Spatial Transcriptomics technologies classified by types and the date they were first published.

Method	Efficiency	Number of gene transcripts	Size resolution	Barcoding Strategy (*in situ*)	Type of Tissue	Commercialized	Advantages	Limitations
**ISS-Based methods**								
ISS	5-30 %	222	Single-cell	SBL	Fresh Frozen	Yes, as Xenium	-Robust detection<br>-High SBR	-Image crowding<br>-Moderate multiplexing capacity
HybISS	Similar to ISS	120	Single-cell	SBH	Fresh Frozen	No	-Enhanced SBR compared to ISS	-Lower number of transcript gene demonstrated than ISS
HybRISS	Increased 5-fold compared to ISS	50	Single-cell	SBH	Fresh Frozen	No	-Enhanced efficiency<br>-Possibility to study more targets than mRNA species	-Lower number of transcript gene demonstrated than ISS
SCRINSHOT	Improved efficiency compared to ISS	29	Single-cell	SBL	Fresh Frozen	No	-Enhanced efficiency<br>-Possibility to study more targets than mRNA species	-Lower number of transcript gene demonstrated than ISS
FISSEQ	(<0,0001%)	Transcriptome wide	Single-cell	SBL	Fresh Frozen	No	-Possibility to conduct *de novo* analyses	-Remarkable low efficiency
ExSeq	60 %	297	Single-cell	SBL/NGS	Fresh Frozen	No	-Enhanced efficiency than FISSEQ<br>-Possibility for *Ex Situ* Analyses	-Increased imaging time
BOLORAMIS	10 % - 35 %	96	Single-cell	SBL	Fresh Frozen	No	-Possibility to study more targets than mRNA species	-Lower number of transcript gene demonstrated than ISS
STAR-MAP	Similar to scRNA-seq	~1000-2700	Single-cell	SEDAL	Fresh Frozen	No	-Possibility to study 3D sections (150 µm thick)	-3D sections limited to 28 genes
**smFISH-Based methods**								
MERFISH	80-95 %	~10,000	Single-cell	N-binary word code decodification	Fresh Frozen FFPE	Yes, as MER-Scope	-High multiplexity capacity<br>-High efficiency<br>-Include Error Correction Scheme	-Low SBR<br>-High number of probes
HCR-seqFISH	~80 %	249	Single-cell	Color-codes associated to mRNA species	Fresh Frozen	No	-High efficiency<br>-Include Error Correction Scheme	-Low SBR<br>-Need for specialized equipment<br>-Moderate multiplexing capacity
SeqFISH+	49 %	~10,000	Single-cell	Color-codes associated to mRNA species	Fresh Frozen	No	-High multiplexity capacity<br>-High efficiency<br>-Include Error Correction Scheme	-High number of probes<br>-Need for specialized equipment<br>-Methodology difficult to handle
Split-FISH	71 %	317	Single-cell	N-binary word code decodification	Fresh Frozen	No	-Enhanced SBR ratios<br>-Include Error Correction Scheme	-Moderate multiplexing capacity
SMI-Nanostring	NA	980	Single-cell	N-binary word code decodification	Fresh Frozen and FFPE	Yes	-Automated system<br>-Possibility to protein co-detection<br>-Include Error Correction Scheme	-Moderate multiplexing capacity
EEL-FISH	~13 %	~2000	Single-cell	N-binary word code decodification	Fresh Frozen	No	-High SBR ratios<br>-Reduced optical crowding	-Lower efficiency compared to other smFISH methods

Table S1. Comparison of ISS and smFISH-based technologies.

Method	Efficiency	Number of mRNA species	-Spot size -Spot-to-spot distance	Covered area	Barcoding Strategy (ex situ)	Type of Tissue	Commercialized	Advantages	Limitations
**Microdissection Technologies**									
LCM	NA	Transcriptome wide	Single-cell resolution	NA	NGS	Fresh Frozen and FFPE	Yes	-Easy implementation (Microdissection microscope) -Available for FFPE	-Limited number of single cells analyzed per tissue
Tomo-seq	NA	Transcriptome wide	Up to 8 μm	NA	NGS	Fresh Frozen	No	-Easy implementation (Sections from a biological system)	-Resolution limited to the area sectioned (lack single-cell resolution)
**Spatial Capturing Technologies**									
ST/Visium	~30.000 UMIs per 100 μm^2 area	Transcriptome wide	-100 μm / 55 μm -200 μm / 100 μm	42.25 mm^2	NGS	Fresh Frozen and FFPE	Yes	-Most available commercial option -Available for FFPE	-Lack single-cell resolution
Slide-seqV2	~550 UMIs per 10 μm^2 area	Transcriptome wide	-10 μm -10 μm	7 mm^2	NGS	Fresh Frozen	No	-High spot size resolution	-Analyses require grouping areas
HDST	~10 UMIs per 2 μm^2 area	Transcriptome wide	-2 μm -2 μm	13.68 mm^2	NGS	Fresh Frozen	No	-High spot size resolution	-Low efficiency -Analyses require grouping areas
DBIT-Seq	~5000 UMIs per 10 μm^2 area	Transcriptome wide	-10 μm or 50 μm -10 μm or 50 μm	1 mm^2 or 25 mm^2	NGS	Fresh Frozen and FFPE	No	-Adapted as a microfluidic system	-Uncovered spaces between squares -Analyses require grouping areas
Seq-Scope	5-25 UMIs per HDMI cluster ~1000 UMIs per 10 μm^2 area	Transcriptome wide	-Submicrometric HDMI cluster -600 nm	0. 2 mm^2	NGS	Fresh Frozen	No	-High spot size resolution -Submicrometric HDMI clusters	-Reduced capturing area -Analyses require grouping areas
Stereo-seq	62 UMIs per DNB cluster (2 μm^2 area) ~1000 UMIs per 10 μm^2 area	Transcriptome wide	-220 nm -600 nm	50 – 200 mm^2	NGS	Fresh Frozen	No	-High spot size resolution -Submicrometric DNB clusters -High capture area (up to 200 mm^2)	-Analyses require grouping areas
Pixel-seq	~1000 UMIs per 10 μm^2 area	Transcriptome wide	~1 μm -Continuous spot-to-spot distance	5-15 mm^2 areas	NGS	Fresh Frozen	No	-High spot size resolution -Developed to be scalable	-Analyses require grouping areas
**Light-based ROI selection technologies**									
GeoMx DSP-Nanostring	~1000 gene transcripts in 400 μm diameter ROIs	Transcriptome wide	ROIs from 10-600 μm	NA	NGS or nCounter	Fresh Frozen and FFPE	Yes	-Easy implementation (Automated system) -Available for FFPE -Possibility for protein codetection	-For whole tissue transcriptomics analyses, laborious manual selection of a limited number of ROIs
Light-seq	~1,000–10,000 UMIs per 10 μm^2 area	Transcriptome wide	Minimum ROI: 2 μm	NA	NGS	Fresh Frozen	No	-ROIs resolution up to 2 μm -Option for sample reutilization	-For whole tissue transcriptomics analyses, laborious manual selection of a limited number of ROIs
**Spatial Cell/Nuclei Dissociation Technologies**									
XYZeq	~1000 UMIs per 500 μm diameter wells	Transcriptome wide	-500 μm wells -500 μm distance	NA	NGS	Fresh Frozen	No	Cells dissociated as barcoded spots	Limited resolution to areas of 500 μm
Sci-space	~2000 UMIs estimated per cell	Transcriptome wide	-73.2 μm -222 μm	NA	NGS	Fresh Frozen	No	Nuclei dissociated as barcoded spots	Limited to nuclear transcriptomics analyses

Table S2. Comparison of microdissection, spatial capture, light-based ROIs selection, and dissociation-based technologies.
